# Draft genome sequence of potential probiotic *Lentilactobacillus hilgardii* S5 isolated from *Nypa fruticans* Wurmb. in Pangasinan, Northern Philippines

**DOI:** 10.1128/mra.01290-25

**Published:** 2026-03-10

**Authors:** Lemuel Ray Balloyan, Jimmbeth Zenila Fabia, Marvielyn Olivar, Grace Sheila Jalani, Peter James Icalia-Gann

**Affiliations:** 1 Center for Cellular and Molecular Medical Research, College of Medicine, Mariano Marcos State University 69374, Batac, Ilocos Norte, Philippines; 2 Research Directorate, Mariano Marcos State University 69374, Batac, Ilocos Norte, Philippines; 3 Department of Biological Sciences, College of Arts and Sciences, Mariano Marcos State University 69374, Batac, Ilocos Norte, Philippines; 4 Department of Biochemistry and Molecular Biochemistry, College of Medicine, Mariano Marcos State University 69374, Batac, Ilocos Norte, Philippines; Fluxus Inc., Sunnyvale, California, USA

**Keywords:** draft genome assembly, probiotics, whole genome sequence

## Abstract

Here, we present the draft genome assembly of a potential plant-based probiotic, *Lentilactobacillus hilgardii* strain S5, isolated from *Nypa fruticans*. This draft assembly provides insights into the genomic characteristics of *L. hilgardii* S5 and will support future *in vitro* studies in its probiotic characteristics.

## ANNOUNCEMENT

Although probiotics are traditionally isolated from dairy products, they can also be sourced from soil, grains, and plant parts, with plant-based probiotics providing a suitable alternative for lactose-intolerant consumers and those seeking more sustainable diets (1–3). Here, a putative probiotic strain, S5, was isolated from *Nypa fruticans* collected from Pangasinan, Northern Philippines (16.00 N°, 120.25 E°). The freshly fermenting sap of the plant was aseptically inoculated onto de Man, Rogosa, and Sharpe (MRS) agar. The plates were incubated at 37°C for 24 hours, after which colony development was examined. Colonies exhibiting morphological characteristics consistent with lactic acid bacteria were subjected to successive streaking to obtain pure isolates. The purified isolate, designated as S5, was preserved in 50% (vol/vol) glycerol and stored at −80°C for long-term preservation.

Strain S5 was cultured in 20 mL MRS broth and incubated with shaking for 24 hours at 37°C prior to genomic DNA extraction. Approximately 1.5 mL of the broth culture was used for genomic DNA extraction using the GF-1 Bacterial DNA Extraction Kit (Vivantis Technologies, Malaysia) following the manufacturer’s protocol. The concentration, purity, and integrity of the extracted DNA were evaluated through agarose gel electrophoresis and quantified using a ScanDrop2 spectrophotometer (Analytik Jena, Germany). High-quality DNA samples were submitted to the DNA Sequencing Core Facility of the Philippine Genome Center for library preparation and sequencing. DNA libraries were constructed using the Nextera XT DNA Library Preparation Kit (Illumina Inc., USA) and assessed for quality using the Agilent TapeStation 2200 System (Agilent Technologies, USA). Whole genome sequencing was subsequently performed on the Illumina MiSeq platform (Illumina Inc.) with a paired-end read of 150 bp.

An initial quality assessment of the raw sequencing reads was performed using FastQC v0.12.1 (4). Raw reads totaled 2,912,530, with the number of obtained bases totaling 421 Mbp. The expected coverage is 130×. Adapter sequences and low-quality bases were trimmed using Trimmomatic v0.40 (5) with a quality threshold of Q20 and a minimum read length of 36 bp. *De novo* genome assembly was carried out using SPAdes v4.2.0 (6), after which the overall quality of the assembled genome was evaluated using QUAST v5.3.0, BUSCO v6.0.0, and CheckM2 v1.1.0 (7–9). Genome annotation was conducted with Bakta 1.11.3 (10), and the resulting annotations were visualized through the Bakta Viewer web interface. For the identification of strain S5, the assembled draft genome was submitted to the Microbial Genome Atlas (MiGA) webserver (11). Default parameters were applied unless specified otherwise.

Here, the assembled genome of strain S5 is 3.1 Mbp in size with a BUSCO score of 99%. Submission of the draft genome to the MiGA database reveals its closest relative as *Lentilactobacillus hilgardii* DSM 20176, with an average nucleotide identity of 98.85%. Genome mining reveals the presence of genes corresponding to probiotic function such as the *dlt* operon (12) and *xylA* (13) for autoaggregation and mucosal persistence, BKEFLP_00857 and BKEFLP_02722 for immunomodulation (14), and BKEFLP_02218 for bacteriocin production. Table 1 provides a summary of the assembly and annotation results.

**TABLE 1:** Assembly and annotation results of strain S5

Assembly statistic	Value for strain S5
Number of contigs	402
Genome size (bp)	3,131,740
Largest contig (bp)	267,483
GC content (%)	39.5
N50	70,491
L50	12
Coding density (%)	84.2
CheckM2 completeness (%)	100
BUSCO (lactobacillaceae_odb12) (%)	99
No. of proteins	2,999
tRNA	67
rRNA	4

## Data Availability

The whole genome data of *Lentilactobacillus hilgardii* S5 can be obtained under the master record accession number JBTNPF000000000.1 in NCBI GenBank. Annotations for each contig are found in JBTNPF010000001–JBTNPF010000402. The BioSample and BioProject accession numbers are SAMN50019342 and PRJNA1293312, respectively. The raw sequences can be accessed in NCBI with the SRA accession number SRR35844807 or with the indicated BioProject number.
